# Ultrasound‐Driven On‐Demand Transient Triboelectric Nanogenerator for Subcutaneous Antibacterial Activity

**DOI:** 10.1002/advs.202204801

**Published:** 2022-11-27

**Authors:** Iman M. Imani, Bosung Kim, Xiao Xiao, Najaf Rubab, Byung‐Joon Park, Young‐Jun Kim, Pin Zhao, Minki Kang, Sang‐Woo Kim

**Affiliations:** ^1^ School of Advanced Materials Science and Engineering Sungkyunkwan University (SKKU) Suwon 16419 Republic of Korea; ^2^ SKKU Institute of Energy Science and Technology (SIEST) School of Advanced Institute of Nanotechnology (SAINT) Samsung Advanced Institute for Health Sciences & Technology (SAIHST) Sungkyunkwan University (SKKU) Suwon 16419 Republic of Korea

**Keywords:** antibacteria, biodegradable, implantable, triboelectric nanogenerator, ultrasound

## Abstract

To prevent surgical site infection (SSI), which significantly increases the rate morbidity and mortality, eliminating microorganisms is prominent. Antimicrobial resistance is identified as a global health challenge. This work proposes a new strategy to eliminate microorganisms of deep tissue through electrical stimulation with an ultrasound (US)‐driven implantable, biodegradable, and vibrant triboelectric nanogenerator (IBV‐TENG). After a programmed lifetime, the IBV‐TENG can be eliminated by provoking the on‐demand device dissolution by controlling US intensity with no surgical removal of the device from the body. A voltage of ≈4 V and current of ≈22 µA generated from IBV‐TENG under ultrasound in vitro, confirming inactivating ≈100% of *Staphylococcus aureus* and ≈99% of *Escherichia coli*. Furthermore, ex vivo results show that IBV‐TENG implanted under porcine tissue successfully inactivates bacteria. This antibacterial technology is expected to be a countermeasure strategy against SSIs, increasing life expectancy and healthcare quality by preventing microorganisms of deep tissue.

## Graphical abstract

A novel strategy of inhibiting microorganisms of deep soft tissue by electrical stimulation is presented through implantable, biodegradable, and vibrant triboelectric nanogenerator for being a potential treatment and prevention method of surgical site infection, especially against antimicrobial resistance, resulting in decreasing the morbidity and mortality rate of patients.

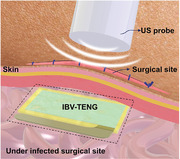

## 1 Introduction

Microorganisms cause surgical site infection (SSI) during the postoperative wound healing process, leading to physical pain and economic burden to patients.^[^ ^1^, ^2^, ^3^ ^]^ SSI can appear in various incisions in the body such as organs, muscles, and skin, which can lead to localized pain in the wound area, abscess formation, organ dysfunction, secondary surgery, or death in some cases.^[^ ^2^, ^4^, ^5^ ^]^ The mortality rate can increase up to 11 times if postoperative SSI happens; furthermore, around half a million SSI cases are reported each year in the United States^[^ ^6^ ^]^ and costing $3–10 billion each year.^[^ ^7^ ^]^ Inhibiting the proliferation of microorganisms is one of the essential ways to prevent SSI.^[^ ^8^, ^9^ ^]^ Since the development of penicillin in 1928, various antibiotics have been used to control microorganisms’ proliferation.^[^ ^10^ ^]^ With the emergence of antimicrobial resistance (AMR), the effectiveness of the existing antibiotics has been compromised, making this one of the most significant health problems of the 21st century.^[^ ^11^, ^12^ ^]^ Furthermore, the emergence of COVID‐19 evoked rising concerns about the limitations of conventional pharmaceutics since highly resistive microbes and variants continue to emerge while developing a suitable vaccine or a cure for them in a short time is difficult. In other words, in the future, microorganisms that cannot be eliminated by drugs, could lead to serious pandemic situations and the concerns have evoked high demands for an alternative solution.^[^ ^13^, ^14^, ^15^ ^]^ Therefore, there is a need to explore a new non‐drug antibacterial technology rather than simply relying on medicines. Various studies have been conducted to control microorganisms through methods such as photodynamic therapy (PDT) and photothermal therapy (PTT).^[^ ^16^, ^17^ ^]^ However, these antimicrobial therapy methods have their shortcomings such as limited selectivity of treating sites, low efficiency, and causing tissue damage due to high temperature and lighting effects.^[^ ^18^ ^]^ Sonodynamic therapy (SDT) is triggered by ultrasound (US), which has many characteristics including deep tissue penetration, non‐invasion and non‐ionization. However, the therapeutic efficiency of SDT is limited by the hypoxic microenvironment of the deep tissues of tumors or infection sites. In addition, sonothermal therapy (STT) is a developed the ultrasonic interfacial engineering of metal/semiconductor (sonosensitizers) interface antibacterial treatment. Nevertheless, the biocompatibility and biodegradability of sonosensitizers need to be developed because of toxicity and biosafety.^[^ ^19^, ^20^, ^21^, ^22^ ^]^ Electrical stimulation (ES) is also one of the non‐drug methods used to inhibit and control the growth of microorganisms.^[^ ^23^, ^24^ ^]^ Various studies have shown that an antibiotic effect can be realized using either direct current (DC) or alternative current (AC).^[^ ^25^, ^26^ ^]^ Nevertheless, the reported strategies primarily apply electrical energy at the suture area close to the superficial wound, limiting their capability to control the microorganisms of deep tissues. Therefore, a novel approach is required to induce low‐voltage electrical energy to prevent SSI inside the body without any tissue damage.

Herein, we present a technology for inhibiting microorganisms under soft tissue using an implantable, biodegradable, and vibrant triboelectric nanogenerator (IBV‐TENG) driven by US to transmit and generate electric stimulation in vitro. The IBV‐TENG produced voltage of ≈4 V and current of ≈22 µA underwater at 3 mm distance from US probe (20 kHz frequency, 2 W cm^−2^ power), which confirmed that up to 100% of *Staphylococcus aureus* and 99% of *Escherichia coli* were eliminated under in vitro environment. Furthermore, ex vivo evaluation indicated inhibiting the bacteria effectively under porcine tissue. Our proposed technology is composed of biodegradable materials so that the device can be degraded, absorbed, and discharged from the body after the antibacterial activity of IBV‐TENG is completed. Furthermore, the degradation rate of the stable encapsulation layer can be accelerated under high ultrasonic intensity. This on‐demand transient property of IBV‐TENG does not require a removal surgery of the device from the body. The advantages of using IBV‐TENG based on transcutaneous US technique are it efficiently removes the bacteria in the surgical site near deep tissue, hence reducing the physical and financial burden of surgical patients.

## 2 Results and Discussion

**Figure 1:**
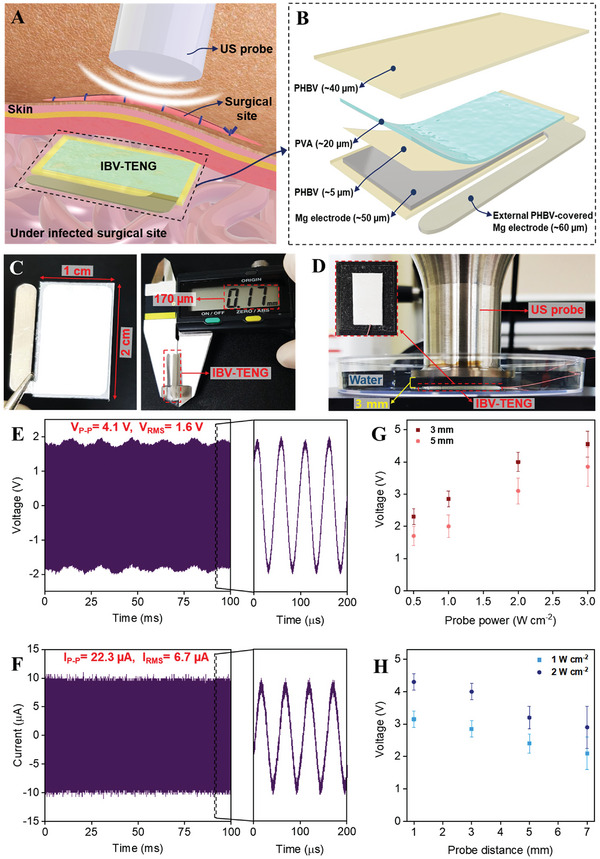
Design and output performance of IBV‐TENG. A) Schematic illustration of IBV‐TENG based on US under the surgical site to prevent SSI by ES. B) The components and structure of IBV‐TENG. C) Front view and thickness of IBV‐TENG. D) Experimental setup for checking output performance of IBV‐TENG underwater. E) Voltage and F) current output signals of IBV‐TENG device measured underwater at 3 mm distance from US probe (20 kHz frequency, 2 W cm^−2^ power). G) Output dependence of IBV‐TENG at different US probe powers (0.5 to 3 W cm^−2^), 3 and 5 mm distance, 20 kHz. H) Output dependence of IBV‐TENG at different US probe distances (1 to 7 mm), 20 kHz, 1 and 2 W cm^−2^.

**Figure** **1**A illustrates the implanted IBV‐TENG under the incision site to prevent SSI by ES. The configuration and components of the device are shown in Figure 1B. The IBV‐TENG was fabricated using biodegradable and biocompatible materials. Poly (3‐hydroxybutyric acid‐co‐3‐hydroxyvaleric acid) (PHBV) was used as both encapsulation and vibrant layer of IBV‐TENG because of its high impermeability, stability, and its triboelectrically positive properties.^[^ ^27^, ^28^ ^]^ Poly(vinyl alcohol) (PVA) was used as a counter friction layer due to its triboelectrically negative property compared to PHBV (Figure S1, Supporting Information).^[^ ^29^, ^30^, ^31^ ^]^ Fourier transform infrared (FTIR) analysis was used to confirm the obtained polymers (Figure S2, Supporting Information). Additionally, Mg foil with a 50 µm thickness was selected as an electrode since Mg has a rapid hydrolysis rate and biocompatibility.^[^ ^32^ ^]^ Mg foil was immersed in the PHBV solution (1%, w/v) to cover for preventing oxidation of Mg foil during the coating process by PVA solution (Figure S3, Supporting Information). The IBV‐TENG was designed as a single electrode mode. Flexible PHBV was utilized as the first layer against the US to promote friction between the triboelectric layers and prevent ultrasound reflection.^[^ ^33^ ^]^ The active area and thickness of the device were 1 × 2 cm^2^ and ≈170 µm, respectively (Figure 1C), and is composed of flexible materials for preventing the discomfort of an unintended wound. Figure 1D and Figure S4, Supporting Information represent to the experimental setup to measure IBV‐TENG output. IBV‐TENG was placed underwater at different distance (1, 3, 5, and 7 mm) from the US probe, 20 kHz frequency, and US power less than 3 W cm^−2^.^[^ ^34^ ^]^ To minimize US energy loss, water was used as a medium for the transmission of ultrasonic waves between the US probe and the IBV‐TENG. In these conditions, the electrical output was a voltage of ≈4 V at 40‐megaohm and a current of ≈22 µA at 1‐ohm (Figure 1E,F). Moreover, the electrical output of IBV‐TENG was investigated under various experimental conditions, such as varying probe power intensity and probe distance. We observed enhancement in electrical output as US probe power intensity was increased (Figure 1G and Figure S7, Supporting Information), while by increasing US probe distance resulted in the electrical output reduction (Figure 1H and Figure S8, Supporting Information).^[^ ^33^ ^]^

**Figure 2:**
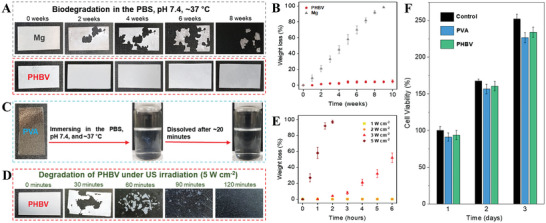
Characterizing biodegradation rate of materials composing the IBV‐TENG. A) Photograph of biodegradation level for every 2 weeks and B) the weight loss rate of Mg foil and PHBV film in PBS (pH 7.4, 37 °C). The thickness of Mg foil and PHBV film were 50 and 40 µm respectively with the same size of 1 × 2 cm^2^. C) Photograph of biodegradation rate of PVA film with 20 µm thickness in the PBS. D) Photograph of biodegradation of PHBV film in PBS under US with 5 W cm^−2^ intensity for every 30 min. E) The weight loss rate of PHBV film under different US intensities. F) Biocompatibility of PHBV and PVA through MTT assay after 1, 2, and 3 days with CRL‐1502 cells.

To remove the IBV‐TENG after completing the role of inhibiting microorganisms deep in the body without an additional surgery, the transient characteristics of materials in the IBV‐TENG must be secured. The biodegradability of the materials (Mg foil, PVA, and PHBV films) used in the fabrication of the IBV‐TENG was experimentally observed after immersed in PBS (pH 7.4, 37 °C).^[^ ^30^ ^]^ The detailed experimental setups are described in the materials and methods section. After immersion, Mg foil undergoes an electrochemical reaction with water, forming a soluble magnesium hydroxide that is fully degraded in ≈8 weeks.^[^ ^35^ ^]^ PHBV film has high stability and low biodegradation rate after 10 weeks, (**Figure** **2**A,B),^[^ ^36^ ^]^ because of its hydrophobicity^[^ ^37^ ^]^ However, PVA film was fully degraded in 20 min because the hydroxyl groups in PVA make strong hydrogen bonds with water molecules (Figure 2C).^[^ ^38^ ^]^ Subsequently, we confirmed that the PHBV film could be degraded under higher US intensity (≥3.0 W cm^−2^). While PHBV film was stable at lower US power (≤2.0 W cm^−2^), degradation started after applying high US intensity of 3 W cm^−2^, and the film was fully degraded at 5 W cm^−2^ within 120 min (Figure 2D,E). This is because the locally strengthened acoustic pressure on the pore inside of the PHBV film promotes the mechanical decomposition, and thus the contact area with water or biofluid is increased, thereby accelerating chemical biodegradation.^[^ ^39^ ^]^ In addition, the biocompatibility of PHBV and PVA was confirmed through in vitro 3‐(4, 5‐dimethylthiazolyl‐2)‐2, 5‐diphenyltetrazolium bromide (MTT) assay tests. The MTT assay results indicate that PHBV and PVA films show high cell viability even after 1, 2, and 3 days (Figure 2F).

**Figure 3:**
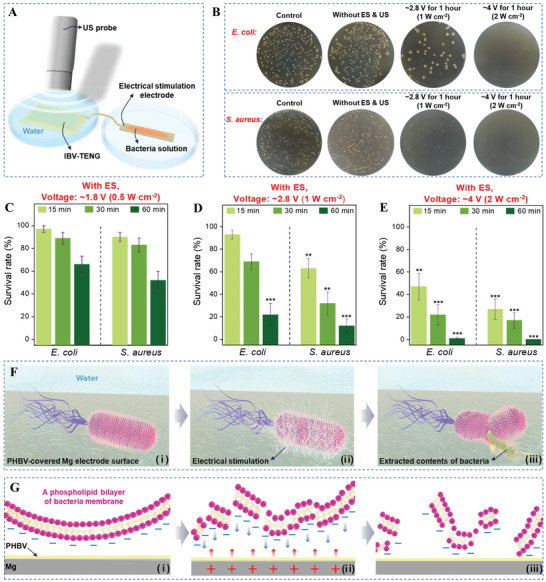
In vitro evaluation of the antibacterial effect of IBV‐TENG by making ES. A) Schematic of the experimental setup evaluating the antibacterial effect of IBV‐TENG underwater at room temperature. US probe was set at 3 mm distance and 20 kHz frequency. The ES was carried out after 15, 30, and 60 min. B) Images of viable bacteria grown after 24 h of culture and C to E antibacterial effect at different conditions as follows; C) inducing ≈1.8 V to bacteria solution, 0.5 W cm^−2^ power of US, D) ≈2.8 V, 1 W cm^−2^, and E) ≈4 V, 2 W cm^−2^. F) Schematic diagram of the destruction of bacteria before and after inducing ES, and G) schematic diagram of the zoomed view of destroying bacteria structure by the mechanism of transferring charged between electrode and bacteria membrane.

The performance of the antibacterial effect of IBV‐TENG was studied through in vitro experiments. As shown in **Figure** **3**A, the IBV‐TENG was attached to the petri dish to be fixed without moving and was placed under the US probe. To identify the real outcomes of ES on the bacteria, the electrode of ES was kept in a separate petri dish next to the fabricated IBV‐TENG to prevent any possible heat effect generated by the US irradiation. The extended Mg electrode was covered with PHBV (≈5 µm) to prevent direct contact with bacteria solution. The bacteria solution (≈10^5^ colony‐forming units per milliliter [CFU mL^−1^], 500 µL) was added to ES part for various treatments. *E. coli* (Gram‐negative) and *S. aureus* (Gram‐positive) were used to investigate the antibacterial activity of IBV‐TENG and to observe the bacterial survival rate after 24 h of culture.^[^ ^40^ ^]^ Figure 3B is a real image of a viable bacteria colony. Figure 3C‐E present quantitative data of bacterial rate under various US intensities. Antibacterial efficiency of IBV‐TENG was observed at different voltage and operation times. Induced ≈4 V of ES by IBV‐TENG for 1 h was the most effective voltage for killing ≈99% of *E. coli* and ≈100% of *S. aureus* (Figure 3E). As shown in Figure 3C–E, bacteria inactivation by ES was much effective on *S. aureus* (Gram‐positive) than on *E. coli* (Gram‐negative). Due to fundamental structural differences in the cell envelope of these two groups of bacteria, Gram‐positive and Gram‐negative, the outer membrane plays an essential role in protecting Gram‐negative microorganisms from the environment and providing an additional stabilizing layer around the cell in comparison to Gram‐positive.^[^ ^41^, ^42^ ^]^ Specifically, after producing the electrical charge by IBV‐TENG, the different electrical potential between the electrode surface and bacteria membrane occurs due to the condition of electrostatic attraction, making disruption in electron transfer of bacterial activities.^[^ ^40^ ^]^ Since the bacteria membrane has negative charge by inducing external electrical charge, the polarity of the membrane surface is changed. This polarization difference destroys the bacteria structure, subsequently, results in leakage and ultimately killing the bacteria (Figure 3F,G).^[^ ^43^, ^44^, ^45^, ^46^, ^47^ ^]^

**Figure 4:**
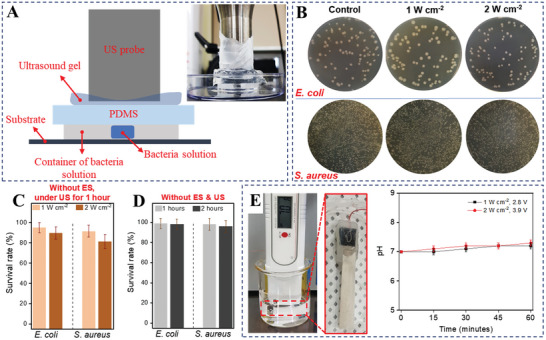
Measurement of antibacterial effect of direct ultrasound inducing. A) Schematic diagram and Real image of experiment setup for checking the antibacterial effect of ultrasound inducing. 1000 µL of bacteria solution was dropped in the plastic petri dish, and then plastic petri dish was closed by a sterilized PDMS (≈200 µm thickness) to prevent direct contact with the ultrasound probe. The distance between ultrasound probe and bacteria solution was fixed at 3 mm, preventing unintended heat from ultrasound probe and evaporation of the bacteria solution during the experiment. B) The image of bacteria colony of *E. coli* and *S. aureus* after inducing ultrasound with 1 and 2 W cm^−2^ power for 1 h. C) Survival rate under direct emission of US without ES, and D) on the PHBV‐covered Mg electrode without ES and US. E) Experimental setup of pH measurement and result when applying electrical stimulation with PHBV‐covered Mg in the 20 mL water. The size of Mg film was 2 × 0.3 cm^2^. For the covering, the Mg electrode emerged in the PHBV solution with 1% w/v. Wire attached on the Mg film by carbon tape then stuck by glue gun on the connection location.

We studied the bacterial survival rate in various conditions to determine the factors involved in survival other than ES. The survival rate was checked under the US with different intensities (from 1 to 2 W cm^−2^) applied to the bacteria sample for 1 h without IBV‐TENG, and there was no significant difference compared to the original rate (**Figure** **4**A–C).^[^ ^48^ ^]^ Furthermore, survival on the PHBV‐covered Mg electrode without ES and US for 2 h; after culture, bacteria grew and kept the proliferation equal the original rate (Figure 4D). Since pH change can significantly affect the bacterial survival rate, the pH was investigated when IBV‐TENG was working under ultrasound. There was no significant difference in pH value during the ES (1 to 2 W cm^−2^) for 60 min, suggesting that the antibacterial activity of IBV‐TENG is pH‐independent (Figure 4E).^[^ ^40^ ^]^

**Figure 5:**
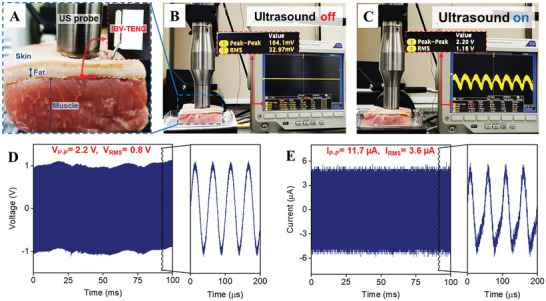
Experimental setup of ex vivo test. A) evaluating the output performance and the antibacterial effect of implanted IBV‐TENG inside porcine tissue under ultrasound. B) The output voltage signal of the IBV‐TENG device when the ultrasound was off. The short output signal is from the environmental noise. C) The output voltage signal of IBV‐TENG on the oscilloscope screen when the ultrasound was on. D) Voltage and E) current output signals of IBV‐TENG at ≈5 mm distance from US probe (20 kHz frequency, 2 W cm^−2^ power).

The antibacterial effect of IBV‐TENG was also evaluated through ex vivo experiment. Initially, to ensure the power generation of IBV‐TENG under ex vivo condition, the IBV‐TENG was implanted inside the porcine tissue under US inducing then the electrical voltage output of the device was measured (**Figure** **5**A–C), and found to be generating a voltage of ≈2.2 V at 40‐megaohm impedance and a current of ≈11.7 µA at 1‐ohm impedance, depth of ≈5 mm and under 2 W cm^−2^ power of US (Figure 5D,E).

**Figure 6:**
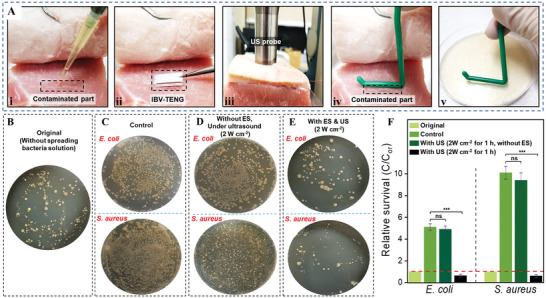
Ex vivo test process for evaluating the antibacterial ability of IBV‐TENG device by making electrical stimulation under ultrasound respectively. A) i) 30 µL of bacteria solution (≈10^5^ CFU mL^−1^) was spread on the porcine tissue with 0.5 cm by 2.5 cm area and kept for 5 h for bacteria proliferation, ii) placing IBV‐TENG on the contaminated part of the porcine tissue. ii) Treatment by making electrical stimulation under ultrasound, iv) Rubbing L‐shape spreader on the contaminated part after treatment, v) Rubbing the L‐shape spreader on the agar plate and locating the agar plate into the incubator for 24 h at 37 °C for culture. B to E) Images of viable bacteria colonies grown after 24 h culture. B) The original colony, related to fresh tissue of pork without applying bacteria solution, C) applying bacteria solution of *E. coli* and *S. aureus* on the tissue without ES and US inducing, D) inducing US (20 kHz, 2 W cm^−2^) without IBV‐TENG, E) inducing ES by IBV‐TENG (2 W cm^−2^). F) Survival rates of *E. coli* and *S. aureus* in different samples.

For studying bacteria survival rate under ex vivo, porcine tissue with fat and muscle layer was selected and 30 µL of bacteria solution (≈10^5^ CFU mL^−1^) was spread on the porcine tissue with 0.5 × 2.5 cm^2^ area.^[^ ^49^, ^50^ ^]^ Then, IBV‐TENG was placed on the contaminated part of the tissue and covered with a skin layer of pork (≈5 mm thickness). The probe of US was fixed just above the skin where we implanted the IBV‐TENG for inducing the US and making ES in the contaminated tissue (**Figure** **6**A). The antibacterial effect was supported by the images of viable bacteria colonies (Figure 6B–E). The induced US was not effective to kill the bacteria under tissue at the initial stage (Figure 6D). The survival rate was reduced by ES via IBV‐TENG, even less than the original rate, that is, over ≈92% of *S. aureus* and ≈86% of *E. coli* were inactivated proportional to the control rate after treatment (Figure 6E,F).

## 3 Conclusion

Recently, biodegradability, comfortability, the antibacterial activity of implanted TENGs with the advantages of simple structure, and high efficiency, have been investigated as a new technology to convert mechanical energy into electricity to be used in the body. Implantable TENGs are growing rapidly based on ultrasound due to the easy and safe mechanical energy transmission in vivo.^[^ ^51^, ^52^ ^]^ Considerably, ES is a well‐known method to inhibit microorganisms in an infected wound. The previous work has already shown that ES in the order of 0.1–10 V cm^−1^ does not damage other human cells.^[^ ^53^ ^]^ In this work, we introduced the US‐driven IBV‐TENG, which can kill the microorganisms by ES under soft tissues to prevent infections. To confirm the antibacterial activity of the US‐driven IBV‐TENG, both in vitro and ex vivo experiments were performed that above 99% and above 89% of the bacteria had been inactivated respectively after inducing the ES while the direct US had no significant effect on microorganisms. After successfully inhibiting the bacteria near the surgical site, the on‐demand IBV‐TENG, which is fully made of biodegradable materials, could be fully degraded in the body, and does not require a removal surgery. This novel strategy of inhibiting microorganisms is expected to be a potential treatment and prevention of SSI, especially against AMRs.

## 4 Experimental Section

### Fabrication of Biodegradable Films

For the fabrication of poly (3‐hydroxybutyric acid‐co‐3‐hydroxyvaleric acid) (PHBV) film: PHBV (98:2) powder (GoodFellow, MW: 410000) was dissolved in chloroform solvent with 5% w/v concentration. The solution was stirred and heated at 90 °C for 3 h on a hot plate. A homogenous solution of PHBV was cast on a glass plate (15 × 15 cm^2^) and subsequently dried at room temperature for 24 h and peeled off. The thickness of the obtained PHBV films was 40 µm. For the fabrication of poly(vinyl alcohol) (PVA) film: PVA powder (Merck, MW: 89000–98000, 99% hydrolyzed) was added in deionized (DI) water with 10% w/v concentration, then the solution was stirred and heated at 120 °C for 2 h on a hot plate to obtain a completely mixed solution of PVA. The PVA solution was spin‐coated on a glass plate at 1000 rpm for 10 s, then dried at room temperature for 24 h. After drying, PVA films were obtained with 20 µm thickness.

### Manufacturing of IBV‐TENG

Mg film with 50 µm thickness was cut by a laser cutter machine. The obtained Mg electrodes had a dimension of 1 × 2 cm^2^. The PHBV solution (5%, w/v) was spin‐coated on the Mg electrode at a speed of 4000 rpm for 10 s and then dried for 6 h at room temperature. Then, the PVA solution (10%, w/v) was spin‐coated on the dried PHBV layer at a speed of 1000 rpm for 10 s and put into the vacuum chamber for 24 h for vaporizing the solvents completely. After drying, a microwire was connected to an uncovered side of the Mg electrode to confirm the output performance of IBV‐TENG. Both sides of the coated Mg electrode were encapsulated with PHBV film (with 40 µm thickness), with the upper side of being used as the friction layer of the IBV‐TENG device. Then, the extra edges of PHBV films that covered the Mg electrode were cut and closed by a needle of hot solder.

### Characterizing Degradation Time of the Materials

The biodegradation time of Mg foil, PHBV, and PVA in the phosphate‐buffered saline (PBS, pH 7.4) at 37 °C was assessed. The thicknesses of Mg foil, PHBV film, and PVA film were 50, 40, and 20 µm, respectively, with an area of 1 × 2 cm^2^ for each. For the Mg foil and PHBV, after completely immersing them in the 20 mL of PBS solution, they were taken out from the container every week, then washed with DI water and dried for 3 h in the oven at 40 °C to observe the biodegradation. Due to the fast‐dissolving PVA film, only the photos of the initial and final dissolution PVA film in the PBS were taken.

### Biocompatibility Test

In vitro biocompatibility assay tests were carried out with CRL‐1502 (skin fibroblast cell) by a MTT method. Those cells (1 × 10^5^ cell mL^−1^) were seeded in a 96‐well plate (100 µL per well) for being incubated at 37 °C for 24, 48, and 72 h. Subsequently, the culture medium was removed in the 96‐well plates (medium suction) and added 50‐µL MTT solution (5 mg/mL) and 50‐µL culture medium to each well. After incubation for 3 h at 37 °C, the MTT solution and culture medium was removed again. The samples were washed using diluted PBS solution and added 150‐µL solubilization solution (dimethyl sulfoxide) to dissolve formazan crystals. Finally, the solution of 100 µL was transferred to other 96‐well plates and measured absorbance to evaluate viability (OD = 570 nm).

### Measurement of Output Performance of IBV‐TENG

Experimental setup of equipment for recording and displaying the output of IBV‐TENG consisted of the oscilloscope (Tektronix DPO3052) with a voltage probe (Tektronix P5100A, 40 µΩ input impendence) and a current probe by using low noise current amplifier (Stanford Research SR570, input impendence of 1 Ω at 500 µA/V calibers). For ultrasound generation, an ultrasonic transductor and generator (Mirae MV100) was employed. Ultrasound and power density were set up and measured by a digital power meter (Yokogawa WT‐1600). The ultrasound probe frame was in cylindrical shape to reduce the noise. The ultrasound probe and device were placed in DI water during the measurements. DI water was used as an impedance matching layer to minimize the ultrasound energy loss during the experiment. The output of IBV‐TENG was demonstrated at the different time scales (2 to 60 min) at 3 mm ultrasound probe distance, 20 kHz frequency with 1 W cm^−2^ power. In addition, the output of IBV‐TENG was measured at the different days in the PBS at 3 mm ultrasound probe distance, 20 kHz frequency with 1 W cm^−2^ power. For checking output dependence of IBV‐TENG at the different ultrasound power, the ultrasound probe was set at 3 and 5 mm distance and frequency of 20 kHz. The setup to study the output dependence of IBV‐TENG at the different ultrasound probe distances (1 to 7 mm) was set at 20 kHz frequency and 1 and 2 W cm^−2^ power density.

### Bacteria Cultures Process

To evaluate the antibacterial effect of IBV‐TENG, Gram‐ negative *E. coli* (ATCC 25922) and Gram‐positive *S. aureus* (ATCC 25923) were obtained from Merck as the experimental samples of model organisms. In brief, the pure bacteria in LB were cultivated overnight in a rotating shaker at 37 °C. Bacteria solutions source of *S. aureus* and for *E. coli* had a concentration of 0.2–1.2 × 10^9^ CFU mL^−1^ that diluted to a volume of 1:9 for three times to concentration of 0.2–1.2 × 10^5^ CFU mL^−1^. 500 µL of each solution of *E. coli* and *S. aureus* were seeded on the PHBV‐covered Mg electrode. After the treatment, the experimental and control bacteria were diluted twice to a volume of 1:9 and 50 µL spread on the agar plates by using the L‐shape spreader for the CFU analysis. The samples were then placed inside the agar culture plate and cultured in the incubator at 37 °C for 24 h. One day before the experiment, 9.2 gr of nutrient agar powder (Merck) was solved in 400 mL DI water, then the bottle of agar solution with the semi‐closed cap was put in an autoclave that uses steam and pressure at 121 °C and 15 PSI for 30 min, for killing the microorganisms on the surface and in agar solution, then placed in the incubator at 75 °C. Solution agar poured in the plastic Petri dish and after drying provided for bacteria culture. The bacterium numbers were measured by Image J software, the bacteria number of original groups was set as A, and the bacteria number of sample groups after treatment was set as B. Finally, the bacterial survival rate (R) is calculated by Equation (1) to evaluate the antibacterial ability of IBV‐TENG.

(1) $$\mathrm{Bacterial}\;\mathrm{survival}\;\mathrm{ratio}\;\left(R\right)=1-\left(A-B/A\times 100\%\right)$$

### In Vitro Antibacterial Test

To observe the antibacterial effect of electrical stimulation from IBV‐TENG without the influence of directly inducing of ultrasonic heat on bacteria solution, the power generation and the electrical stimulation were divided into two parts. The device was stuck in the two plastic petri dishes by double side tape, once the petri dish contained encapsulated IBV‐TENG, and the next petri dish contained the PHBV‐covered Mg film with PHBV (≈5 µm, for prevention of reaction of Mg with bacteria solation), which is bacteria solution location. Encapsulated IBV‐TENG and PHBV‐covered Mg electrode were connected by a microwire. Encapsulated IBV‐TENG was in the water during the experiments at 3 mm distance from the ultrasound probe with 20 kHz frequency to make electrical stimulation for killing the bacteria in the next petri dish which has PHBV‐covered Mg electrode and 500 µL of bacteria solution. The images of bacteria culture were then recorded and analyzed and calculated the number of viable bacteria grown on different samples after 24 h of culture. The antibacterial ability of IBV‐TENG was studied on Gram‐negative (*E. coli*) and Gram‐positive (*S. aureus*) and applied different voltages to kill the bacteria. Ultrasound power was changed at a constant distance to obtain the different voltages.

### Ex Vivo Antibacterial Test

The antibacterial ability of IBV‐TENG was evaluated from ex vivo. In the beginning, the fresh soft tissue of pork with fat layer and skin provided was cut into parts at same condition and size, thickness of ≈4 cm and 8 × 8 cm^2^ dimension. 30 µL of bacteria solution (≈10^5^ CFU mL^−1^) was dropped on the particular place of fresh tissue with 0.5 × 2.5 cm^2^ dimension for 5 h, then IBV‐TENG was placed on the contaminated part of the tissue and covered by skin layer of pork (≈5 mm thickness). The probe of ultrasound was fixed on the skin where IBV‐TENG is implanted, for inducing ultrasound vibration and making electrical stimulation on the contaminated tissue. After treatment, an L‐shape spreader was rubbed on the contaminated part, culturing the bacteria on the agar plate and the samples were placed inside an incubator at 37 °C for 24 h.

### Statistical Analysis

Statistical analysis was referenced in detail according to checklist: https://www.advancedsciencenews.com/road‐better‐data‐presentation‐dos‐donts/. Data of all experiments were evaluated as mean values ± standard deviation (SD) of at least three tests from a representative experiment (*n* = 3 independent samples per group). The error bars mean the standard deviations. Statistical significance of the variance was evaluated by the GraphPad prism using the two‐way analysis of variance (ANOVA) program using the Holm–Sidak method. Values of *p < 0.05, **p < 0.01, and ***p < 0.001 are considered statistically significant. The “ns” means no significant difference.

## Conflict of Interest

The authors declare no conflict of interest.

## Supporting information

Supporting information

## Data Availability

The data that support the findings of this study are available from the corresponding author upon reasonable request.
