# Supplementary material for: Ultrasound‐Driven On‐Demand Transient Triboelectric Nanogenerator for Subcutaneous Antibacterial Activity
Source: Adv Sci (Weinh). 2022 Nov 27;10(3):2204801. doi: 10.1002/advs.202204801 (PMC9875681; doi:10.1002/advs.202204801)
Supplement: Supplementary file 1 — Supporting information [file ADVS-10-2204801-s001.pdf]

ADVANCED SCIENCE
Open Access

Supporting Information

for *Adv. Sci.*, DOI 10.1002/advs.202204801

Ultrasound-Driven On-Demand Transient Triboelectric Nanogenerator for Subcutaneous Antibacterial Activity

*Iman M. Imani, Bosung Kim, Xiao Xiao, Najaf Rubab, Byung-Joon Park, Young-Jun Kim, Pin Zhao, Minki Kang and Sang-Woo Kim**

Supporting Information

Ultrasound-Driven On-Demand Transient Triboelectric Nanogenerator for Subcutaneous Antibacterial Activity

*Iman M. Imani†, Bosung Kim†, Xiao Xiao, Najaf Rubab, Byung-Joon Park, Young-Jun Kim, Pin Zhao, Minki Kang, Sang-Woo Kim**

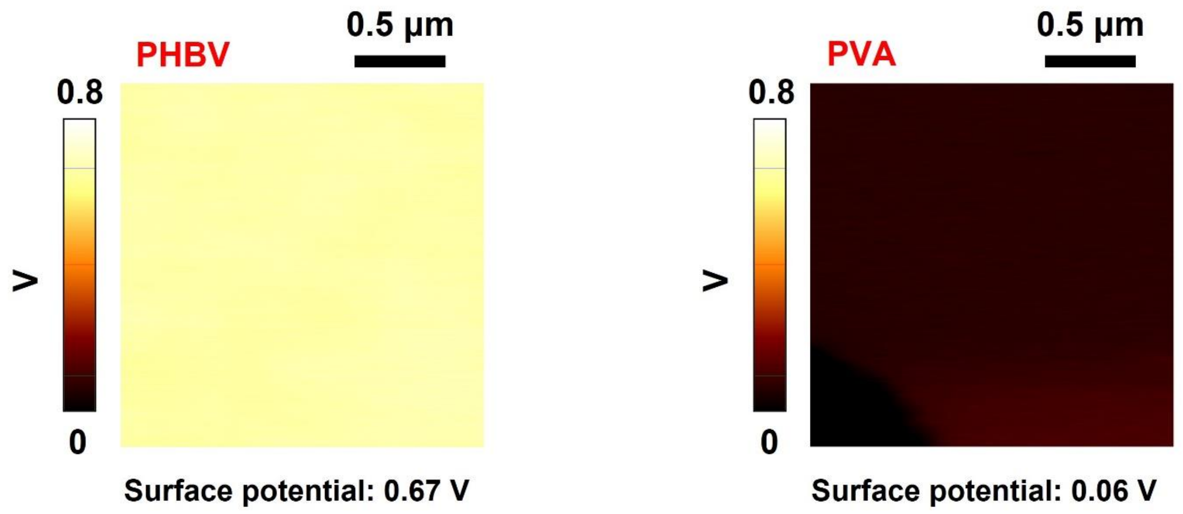

Figure S1. The surface potential of PHBV and PVA films measured by Kelvin probe force microscopy (KPFM). These are the surface potentials of PHBV and PVA films that were used as friction layers in the IBV-TENG device. The surface potential of PHBV film was approximately 0.67 V, which is higher than the surface potential of PVA, 0.06 V. Therefore, when the two materials are frictional, positive charge moves on the surface of PHBV, and negative charge moves on the surface of PVA. These surface potentials were measured by KPFM measurements using AFM (Park Systems, XE-100) with Pt/Cr-coated silicon tips (Multi75E-G, BudgetSensors). The scan area was 2 μm × 2 μm, and the scan rate was 0.5 Hz. The KPFM measurement was conducted under the condition of 2 V AC voltage signal with 17 kHz frequency, which is applied on the samples by a lock-in amplifier.

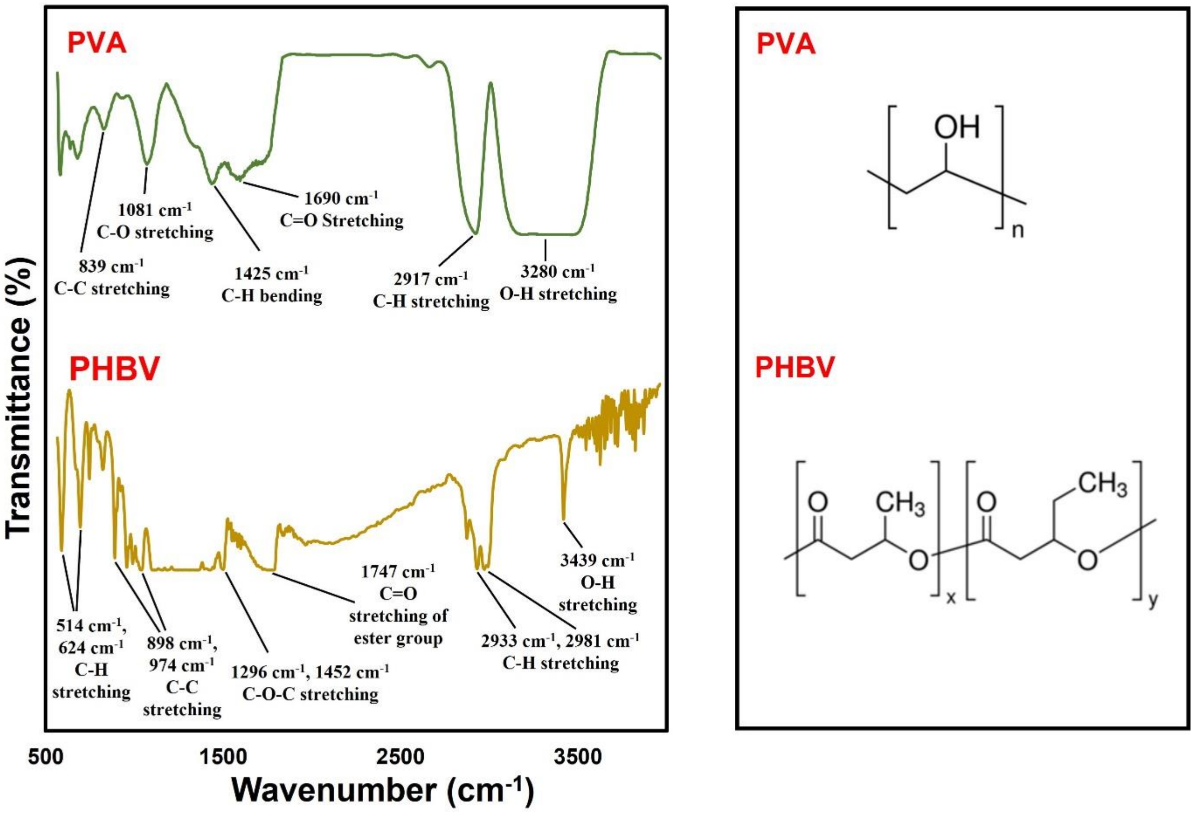

Figure S2. Chemical structure and FTIR spectroscopy spectra to identify the synthesized polymers (PHBV, PVA).

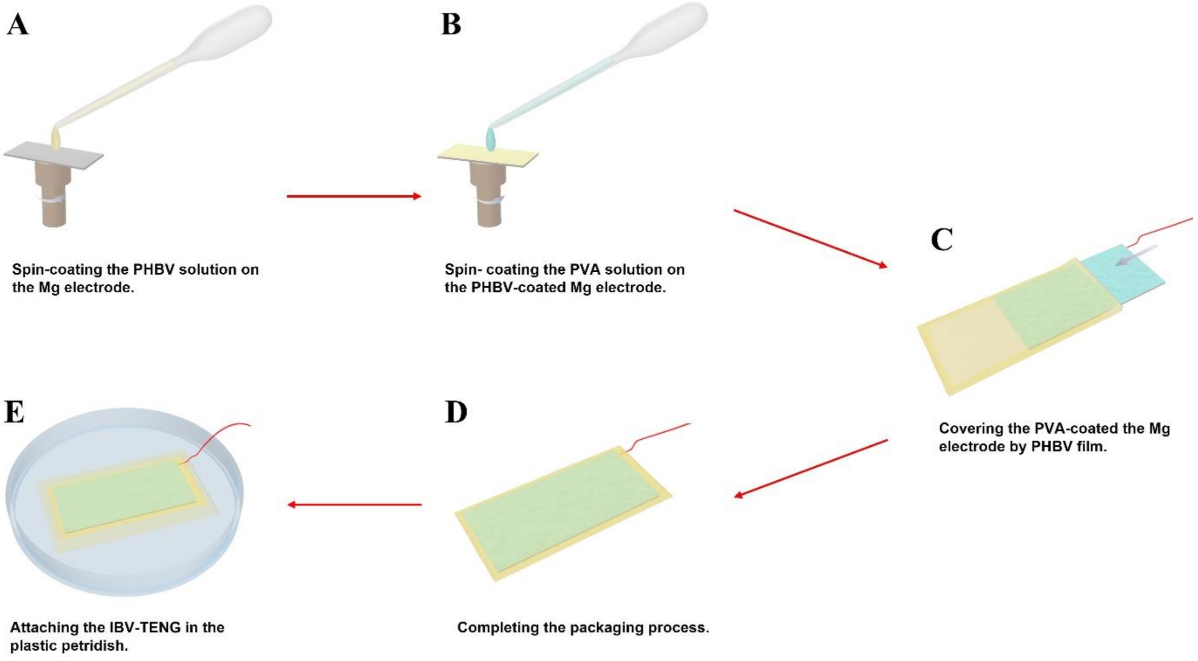

Figure S3. The manufacturing process of IBV-TENG device. A) Initially, PHBV solution (5%, w/v) was spin-coated on the Mg electrode (dimension of 1 × 2 cm^2) at 4000 rpm for 10 seconds, then dried at room temperature for 6 hours. This PHBV layer (5 µm thickness) was used as a protective layer to prevent the Mg electrode from direct contact with PVA solution, which oxides the magnesium. B) Subsequently, PVA solution (10%, w/v) was spin-coated on the PHBV layer at 1000 rpm for 10 seconds, then dried in a vacuum chamber at room temperature for 24 hours. The thickness of the PVA layer was 20 µm. C) The PVA-coated Mg electrode was covered with a PHBV film to be used as both encapsulating and friction layer, D) and then the extra edge was cut and closed by a needle of hot solder. E) The IBV-TENG device was attached at the bottom of the plastic petri dish by double side tape for fixing under the water by necessity.

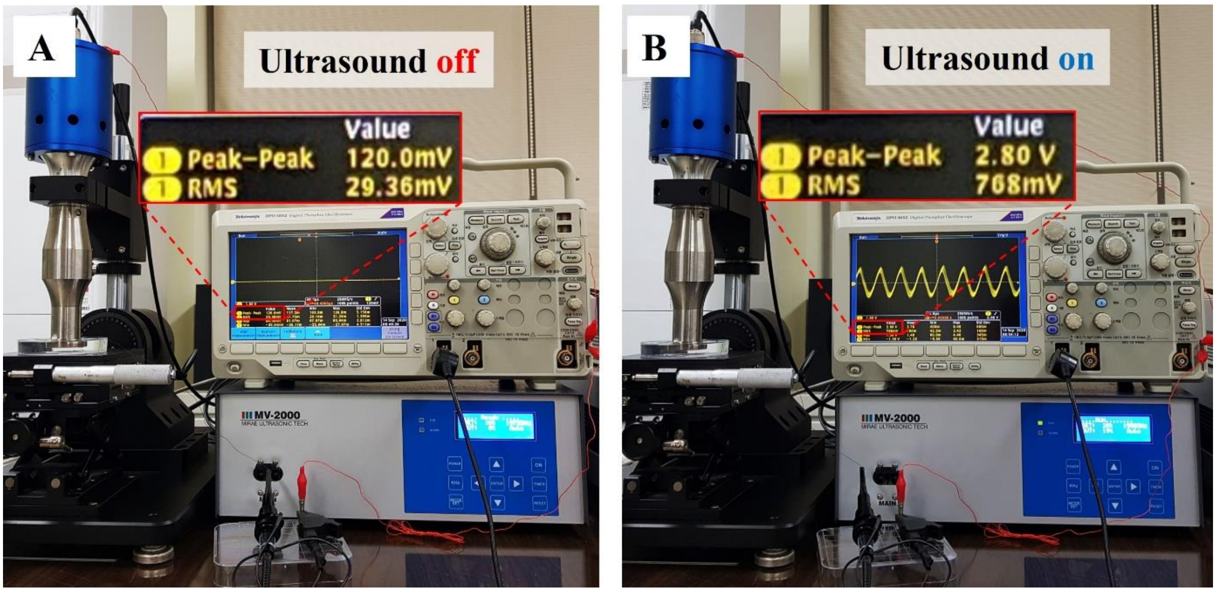

Figure S4. Experimental setup of equipment for checking the output of IBV-TENG under ultrasound probe in water. A) The output voltage signal of the IBV-TENG device when the ultrasound was off. The short output signal is from the environmental noise. B) The output voltage signal of IBV-TENG on the oscilloscope screen when the ultrasound was on. IBV-TENG was placed underwater at 3 mm distance from the ultrasound probe which was set at 20 kHz frequency and 1 W cm^{-2} intensity level.

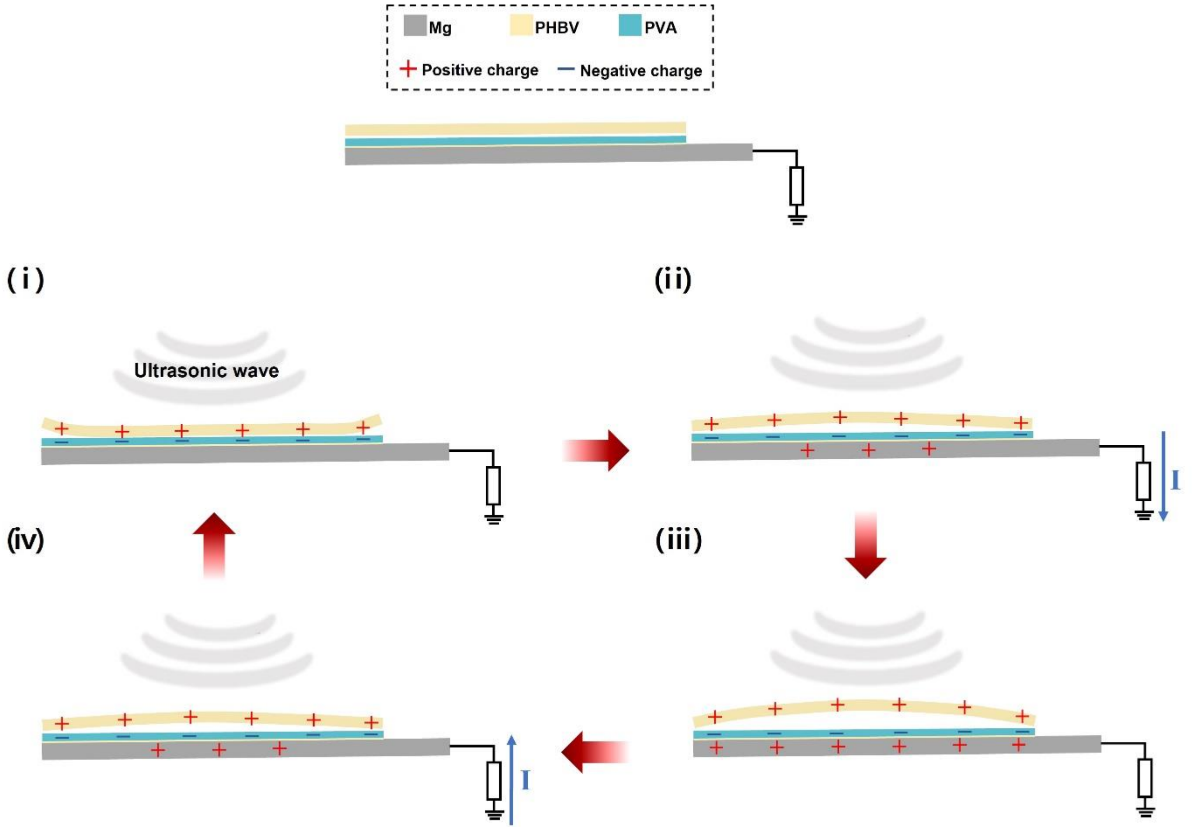

Figure S5. Charge generation of IBV-TENG under ultrasound. As shown, the phenomena of the charge generation mechanism of IBV-TENG by inducing ultrasonic vibrations (ultrasound) were illustrated in four steps. In the beginning, PHBV and PVA layer contact with each other by applying the ultrasound wave, generating equal opposite triboelectric charges on the surface. As shown in Figure S3, due to different surface potentials, the positive charges are generated on the surface of PHBV, while negative charges are generated on the surface of PVA. When the PHBV layer moves away from the surface of the PVA as a result of the pressure gradient of the ultrasonic wave, the electron will move through the electrode to the external circuit to balance the potential difference between the electrode and the ground; consequently, the charge will be obtained in the eternal circuit. When the PVA and PHBV layers move towards each other, the contrary direction of the charge will find a flow in the external circuit until the two layers contact completely, so this process repeats and continues by keeping the inducing ultrasonic wave.

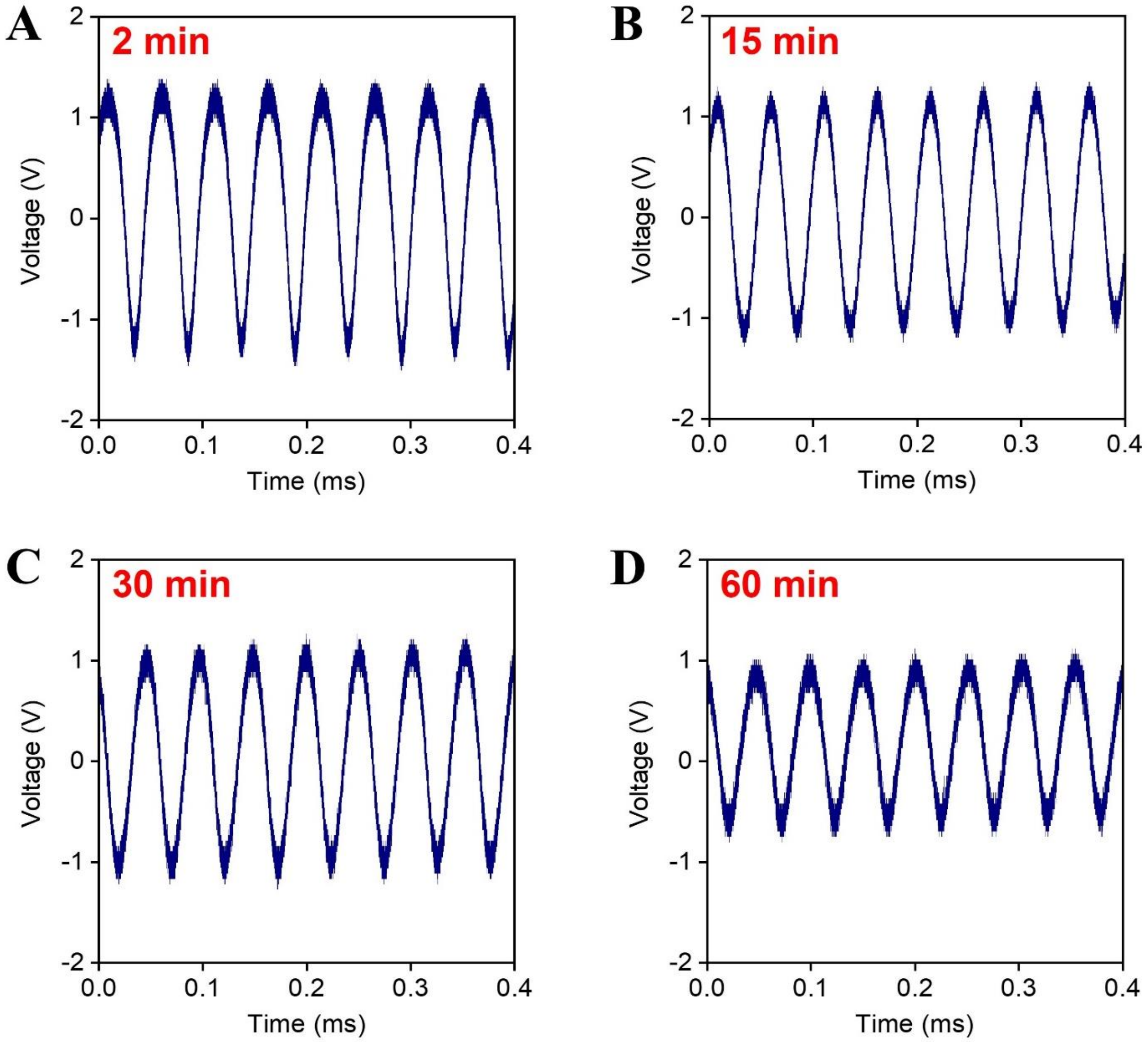

Figure S6. The output signal of IBV-TENG at different times under ultrasonic vibrations. The output voltage signals of IBV-TENG when applying ultrasound for a certain period from A) 2 minutes to D) 60 minutes. The IBV-TENG was placed underwater at 3 mm distance from the ultrasound probe, which was set at 20 kHz frequency and 1 W cm^{-2} power intensity.

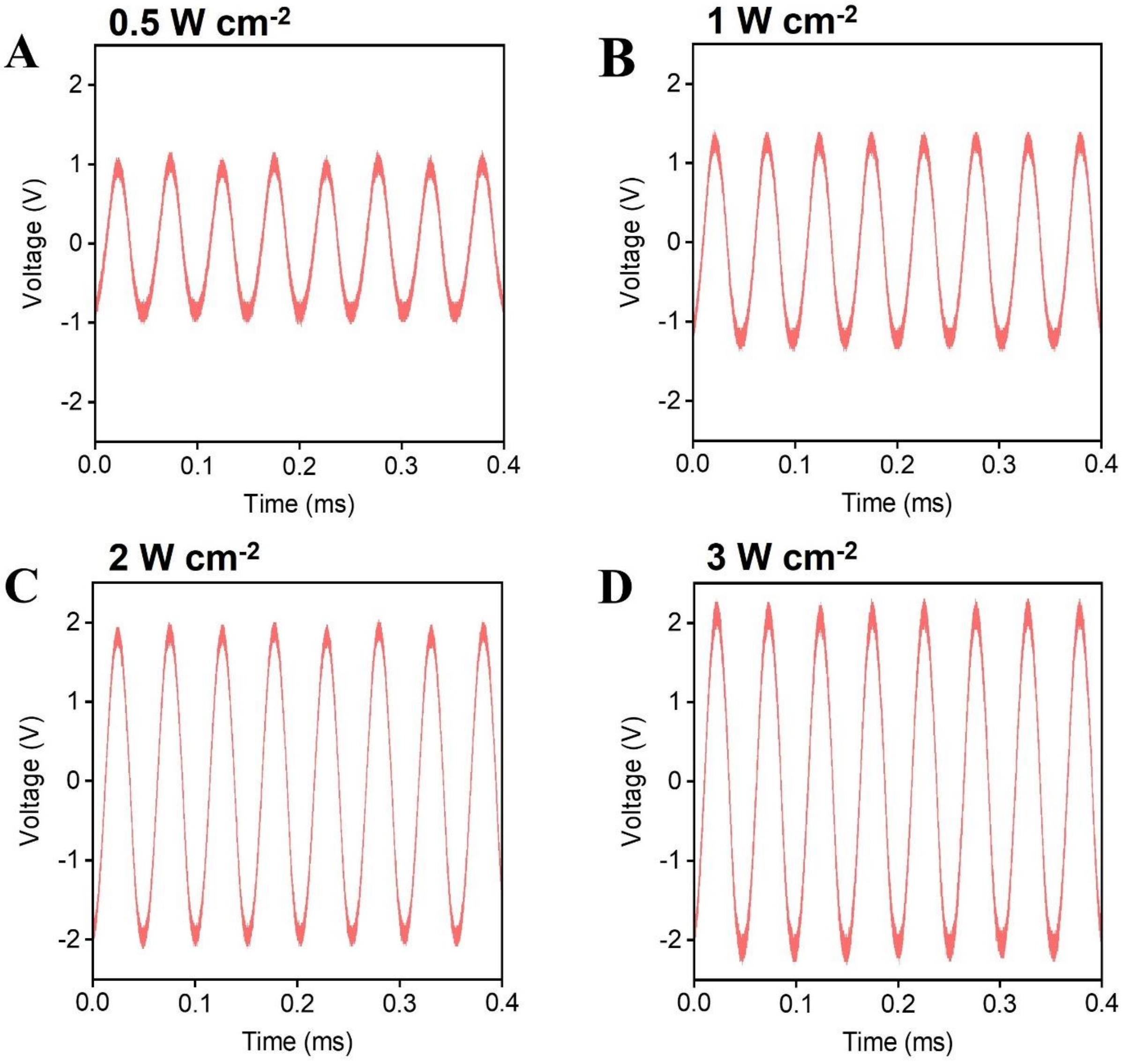

Figure S7. The output voltage signal of IBV-TENG under different ultrasound intensities. The output voltage of IBV-TENG under the different ultrasound intensity from A) 0.5 W cm^{-2} to D) 3 W cm^{-2}. The IBV-TENG was placed underwater at 3 mm distance from the ultrasound probe at 20 kHz frequency.

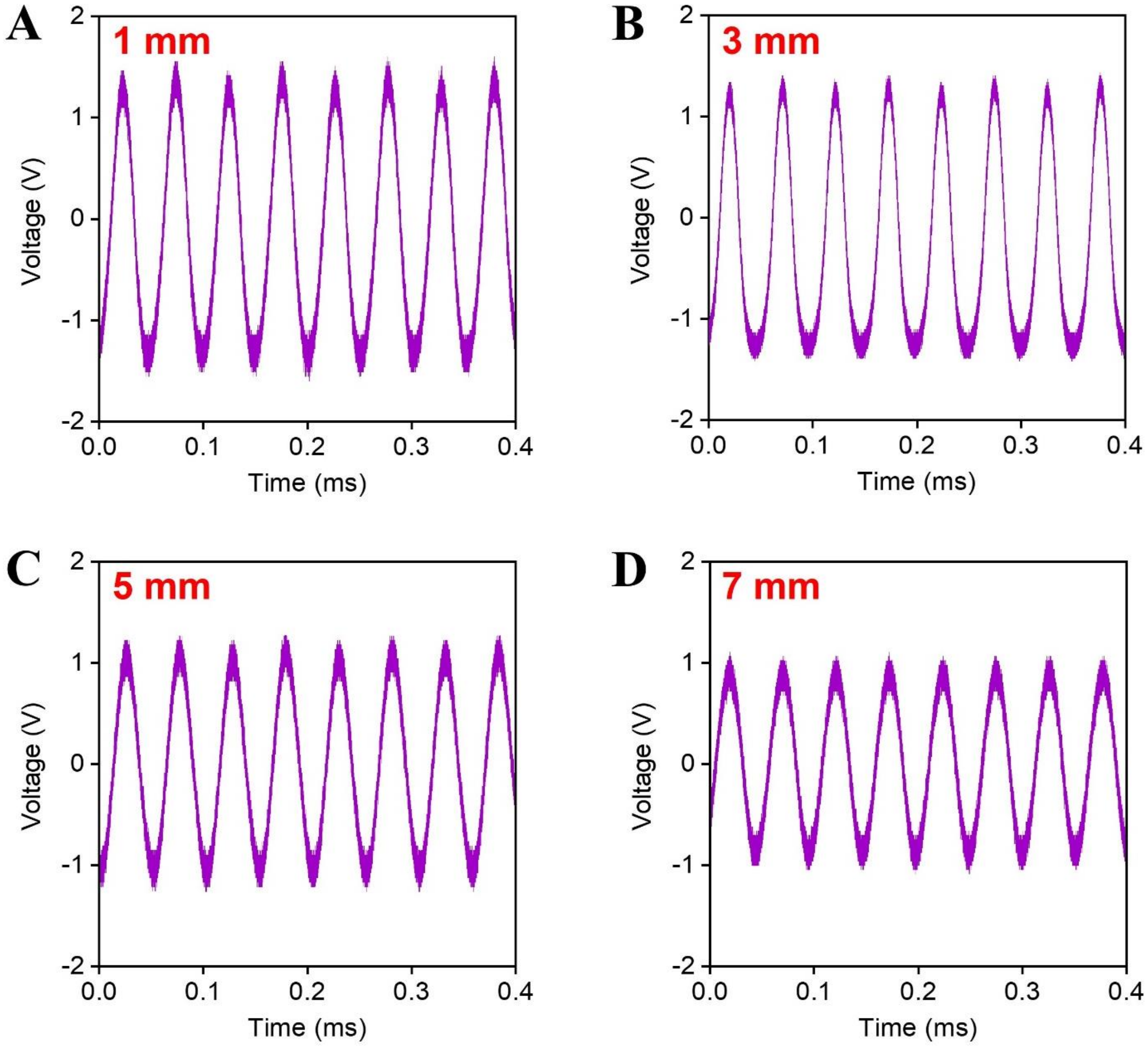

Figure S8. The output voltage signal of IBV-TENG under different ultrasound probe distances. The output voltage of IBV-TENG devices under different ultrasound probe distances from A) 1 mm to D) 7 mm above the device. The IBV-TENG was placed underwater, and the ultrasound probe was set at 20 kHz frequency and 1 W cm^{-2} intensity level.

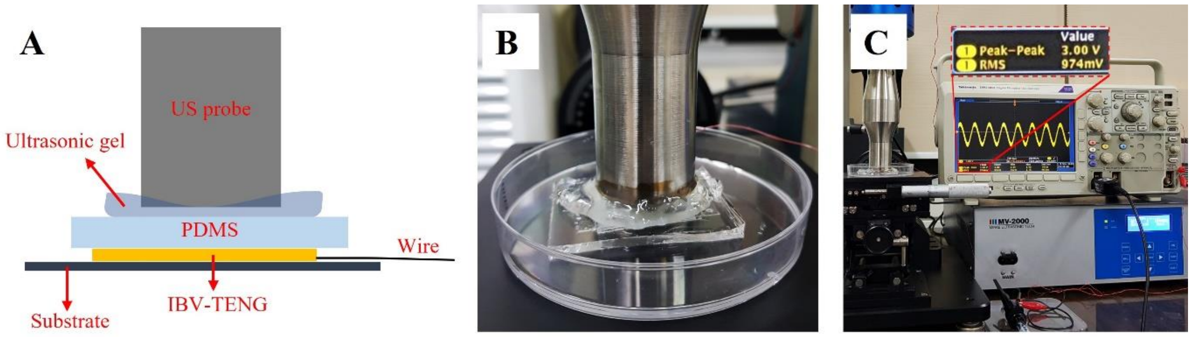

Figure S9. Experimental setup for checking the output of IBV-TENG when using PDMS and ultrasonic gel as a matching layer. A) Schematic diagram and B) real image of experiment setup for checking the IBV-TENG output under PDMS film and ultrasound gel. C) Voltage signal as shown in the oscilloscope screen. The frequency of the ultrasound probe was set at 20 kHz with 1 W cm^{-2} power, and the thickness of PDMS was 3 mm.

References

[1] R. A. Gross, B. Kalra, *Science* **2002**, 297, 803.

[2] A. Musetti, K. Paderni, P. Fabbri, A. Pulvirenti, M. Al-Moghazy, P. Fava, *J. Food Sci.* **2014**, 79, E577.

[3] R. Machatschek, A. Lendlein, *J. Control Release* **2020**, 319, 276.

[4] R. Das, A. Chanda, *Nano-size Polymers*, Springer, **2016**, 283.

[5] K. Leja, G. Lewandowicz, *Pol. J. Environ. Stud.* **2010**, 19, 255.

[6] T. L. A. Montanheiro, F. R. Passador, M. P. Oliveira, N. Durán, A. P. Lemes, *Mater. Res.* **2016**, 19, 229.

[7] D. A. Robinson, R. W. Griffith, D. Shechtman, R. B. Evans, M. G. Conzemius, *Acta Biomater.* **2010**, 6, 1869.

[8] D. Freebairn, D. Linton, E. Harkin-Jones, D. S. Jones, B. F. Gilmore, S. P. Gorman, *Expert. Rev. Med. Devices* **2013**, 10, 85.

[9] J. C. Lagier, S. Edouard, I. Pagnier, O. Mediannikov, M. Drancourt, D. Raoult, *Clin. Microbiol. Rev.* **2015**, 28, 208.

[10] Y. Zhang, Z. Zhou, L. Sun, Z. Liu, X. Xia, T. H. Tao, *Adv. Mater.* **2018**, 30, 1805722.

[11] S. Gratz, H. Mykkanen, H. El-Nezami, *J. Food Prot.* **2005**, 68, 2470.
